# A retrospective clinical and microbial analysis of 32 patients with bilomas

**DOI:** 10.1186/s12876-019-0968-2

**Published:** 2019-04-04

**Authors:** S. Würstle, A. Göß, C. D. Spinner, W. Huber, H. Algül, C. Schlag, R. M. Schmid, A. Weber, A. Obermeier, J. Schneider

**Affiliations:** 1 Klinikum rechts der Isar, Technische Universität München, Innere Medizin II, Ismaningerstrasse 22, 81675 Munich, Germany; 2 Klinikum rechts der Isar, Technische Universität München, Klinik für Orthopädie und Sportorthopädie, Implantat-assoziierte Infektforschung, Munich, Germany

**Keywords:** Biloma, Bilioma, Bile, Pathogen spectrum

## Abstract

**Background:**

Bilomas are defined collections of bile fluids mainly caused by iatrogenic injuries of the bile duct system. Owing to the infrequency of this disease, studies addressing bilomas are rare.

**Methods:**

By using an endoscopic database, this retrospective study identified 32 patients with bilomas treated between 2004 to 2015, in order to analyse aetiology, clinical presentation, spectrum of pathogens, and resolution rate of bilomas.

**Results:**

65.6% of the study population (21/32) developed bilomas after surgery and 21.9% (7/32) after endoscopic retrograde cholangiography (ERC). Icterus, fever, and abdominal pain were the leading symptoms. 93.9% (46/49) of microbiological bile cultures revealed a positive microbiology. The predominant microorganisms were the group of *Enterobacteriaceae* (43.0%, 52/121), followed by *Enterococcus* spp. (32.2%, 39/121), and *Candida* spp. (9.1%, 11/121). Multiresistant bacteria like *Enterobacteriaceae* were isolated from one quarter of all patients. Single or multimodal treatment resulted in an overall complication rate of 4.8% (9/188). Clinical follow-up analysis showed a complete resolution rate of 78.3% for interventional therapy and 80% in the non-interventional group.

**Conclusions:**

Pathogen spectrum of bilomas mainly comprises the group of *Enterobacteriacae* and *Enterococcus* spp., with a high proportion of multiresistant bacteria. Different interventional approaches are available for biloma drainage, which seem to be safe and effective for most patients.

**Trial registration:**

German Clinical Trials Register DRKS00015208, retrospectively registered.

## Background

Bilomas are encapsulated intra- or extrahepatic collections of bile fluid caused by an injury of the bile duct resulting in biliary leakages. [1]. Biliary leakages of the bile duct often close spontaneously, however major leakages may result in bilomas [2]. The alkaline characteristic of bile triggers inflammation in the surrounding parenchyma leading to adhesion and demarcation of the biliary collection [3]. Genesis of biloma are mainly iatrogenic injury of the bile duct system [4], whereas spontaneous bilomas are very rare and mostly associated to choledocholithiasis [5]. Surgical interventions like cholecystectomy or liver transplantation are the most common cause of bilomas [4]. In case of infection, the differentiation of bilomas from liver abscesses is challenging, although abscesses are less caused by interventions and tend to be more encapsulated compared to bilomas [6]. Symptoms of biloma include jaundice, abdominal pain, loss of appetite, nausea and vomiting [3]. Antibiotics in case of infection and drainage of bilomas, i.e. using percutaneous transhepatic cholangiodrainage, endoscopic retrograde cholangiography (ERC) or CT-, or sonographic-guided puncture are the mainstay of treatment. In case of asymptomatic, uncomplicated bilomas a wait-and-see approach may also result in complete resolution of bilomas. To date, clinical knowledge of biloma is mainly based on case reports and small series studies [5, 7–10]. Major focus of the current trial was to analyse aetiology, clinical presentation, the pathogen spectrum, and treatment of patients with bilomas.

## Methods

### Patients

A total of 32 patients of the Klinikum rechts der Isar, Technische Universität München treated between 2004 to 2015 were retrospectively included. Patients were recruited as follows: First, all patients who underwent ERC or percutaneous transhepatic cholangiodrainage (PTCD) were identified using an endoscopic database. In the next step the findings of ERC/PTCD were screened for bilomas. Main/side diagnosis, clinical and laboratory parameters as well as status of microbiology (positive/negative/not performed) were reviewed for all patients with one or more bilomas.

Following inclusion criteria were defined:

- Presentation of a bile fluid collection after injection of contrast agent into the biliary system via ERC/PTCD
- Typical medical history like ERC, trans arterial chemoembolization (TACE), hepatobiliary surgery, photodynamic therapy or liver transplantation

Patients were excluded from the study when patient records were incomplete.

The term infected biloma was applied in case of fever (> 38.5 C) with CRP-elevation (> 5 mg/dl) or leucocytosis (> 12.0 G/l) and no indications for other focus of infection. Referring to the Standard Operating Procedure (SOP) of our department of gastroenterology, antibiotic therapy was initiated after bile collection, provided that the patient was not septic.

### Drainage methods and collection of bile fluid cultures

Bile was collected by endoscopic retrograde or percutaneous transhepatic biliary or CT−/sonographic-guided access. ERC was performed with a standard videoduodenoscope (TJF-160 VR). In case of difficult bile duct cannulation, precut techniques such as transpancreatic or needle knife precut sphincterotomy were used. Subsequently, a polyethylene-stent was either placed into the biloma or close to the biliary leakage. The point of entry of the PTCD was determined by using ultrasound guidance. Subsequently, a Chiba needle was inserted into the biliary system. Contrast agent was injected and the biliary system was outlined on X-rays. Finally, a Yamakawa drain was inserted using the Seldinger technique. In case of failure of these techniques, Rendezvous intervention was performed as a salvage technique combining an endoscopic with a percutaneous transhepatic approach. Sonographic or CT-guided drainage was also conducted by using Seldinger Technique. Bile was collected in a sterile 10 mL syringe after puncture of the bile duct system.

### Microbiological processing

Bile was injected into aerobic/anaerobic blood culture flasks and subsequently incubated for 5 days at 37 °C. Each flask was subcultivated under aerobic (chocolate agar in 5% CO2) and anaerobic conditions (Schaedler anaerobic agar) at the end of the incubation period for control and to exclude failure of automatic detection of the BacTec system (BD Becton Dickinson, Franklin Lakes, USA). If bacterial growth was detected in a culture flask, 50–100 μL of bile was transferred onto solid culture media (Columbia sheep blood agar, chocolate agar, MacConkey agar, Schaedler anaerobic agar, Schaedler KV anaerobic agar and Sabouraud agar) and subsequently cultured for at least 48 h at 37.8 °C.

### Microbial identification and antibiotic susceptibility testing

Microbial identification was performed by biochemical testing systems (ATB, API and VITEK system, bioMerieux, Nürtingen, Germany) or matrix-associated laser desorption/ionization-time of flight (MALDI-TOF, Bruker Corporation, Billerica, USA). An automated microdilution system (VITEK system, bioMerieux, Nürtingen, Germany) or standardized discs were used for antimicrobial susceptibility testing. Antimicrobial susceptibility testing was interpreted by using CLSI and EUCAST methodologies.

### Statistical analysis

The categorical variables are expressed as number and percentage and the continuous variables as median and range. Fisher’s two-sided exact test was used to compare qualitative parameters and Wilcoxon’s rank-sum test for quantitative parameters. *P*-values less than *P* = 0.05 were considered statistically significant. Statistical analyses were performed with SPSS Statistics (version 24, SPSS Inc., Chicago, USA) and R (version 3.4.3; R Foundation for Statistical Computing, Vienna, Austria).

## Results

### Baseline characteristics

In total 32 patients were included in the study, see also Table 1. The median age was 61 years (range 18–83 years). 65.6% (21/32) underwent surgery before developing bilomas including liver transplantation (7/32, 21.9%), cholecystectomy (6/32 18.8%) and partial liver resection (5/32, 15.6%). Non-surgical aetiology of bilomas were due to ERC (21.9%, 7/32), photodynamic therapy (6.3%, 2/32), or transcatheter arterial chemoembolization (TACE, 3.1%, 1/32). One patient developed a biloma based on a stab wound injuring the right lobe of the liver, the small intestine, and the transverse colon. In total, the median interval from triggering event to diagnosis of biloma was 31 days (range 3–872 days). On subgroup analysis, median time to diagnosis was 36 days (range 6–872 days) for biloma with a surgical aetiology and 31 days (3–94 days) for bilomas with non-surgical aetiology.

**Table 1:** Baseline characteristics

Variables	Absolute frequency	Percentage
Number of patients	32	100.0%
male	19	59.4%
median age in years (range)	61 (18–83)	
Aetiology		
Surgery	21	65.6%
ERC	7	21.9%
Photodynamic therapy	2	6.3%
TACE	1	3.1%
Trauma	1	3.1%
Clinical symptoms	22	68.8%
Icterus	20	62.5%
Fever	12	37.5%
Abdominal pain	11	34.4%
Multiple biloma	11	34.4%

### Characteristic of bilomas

In total, 54 bilomas were detected. Most patients had only one biloma (21, 65.6%), whereas 11 (34.4%) patients presented multiple bilomas including two patients with 4 and two patients with 5 bilomas. Aetiology of multiple biloma was both surgery (54.5%, 6/11) and non-surgery (45.5%, 5/11). The median diameter of a biloma was 5.4 cm (range 2–19.5 cm). Asymptomatic bilomas were slightly smaller with a median of 5 cm than symptomatic bilomas (5.7 cm, *P* = 0.979, Wilcoxon test). Most bilomas were localized intrahepatic (86.7%).

### Clinical presentation

68.8% (22/32) presented clinical symptoms. Icterus (20/32, 62.5%), fever (12/32, 37.5%) and abdominal pain (11/32, 34.4%) were the leading symptoms. Patients with symptomatic bilomas stayed significantly longer in hospital (median 34 days, range 4–108) than asymptomatic patients (median 9 days, range 2–37, *P* = 0.010). Furthermore, patients with multiple bilomas (9/11, 81.8%) presented more frequently clinical symptoms compared to patients with only one biloma (13/21, 61.9%, *P* = 0.4250). 10 patients had infected bilomas according to the above-mentioned criteria. Table 2 compares laboratory parameters between patients with and without infected biloma: GOT (glutamic oxaloacetic transaminase) was significantly elevated in patients with infected bilomas, whereas no significant difference was seen concerning cholestasis parameters like bilirubin or gamma-GT.

**Table 2:** Laboratory parameters of infected and non-infected bilomas (median, range)

Laboratory parameters	Infected bilomas	Non-infected bilomas	P-value
Bilirubin in mg/dl	1.6 (1.1–4.0)	1.1 (0.3–29.2)	0.2119
Gamma-GT in U/l	465 (93–1125)	274 (35–1228)	0.2619
GOT in U/L	104 (63–211)	51 (19–174)	0.0043
GPT in U/L	95.5 (25–194)	54 (7–276)	0.0852

**Table 3:** Spectrum of pathogens of biloma

Microorganisms	Absolute frequency	Percentage
all microorganisms	121	100.0%
Aerobe gram-positive bacteria	48	39.7%
Enterococcus spp.	39	32.2%
Enterococcus faecium	27	22.3%
Enterococcus spp. susceptible to Ampicillin	12	9.9%
Staphylococcus spp.	5	4.1%
Coagulase negative staphylococcus	4	3.3%
Staphylococcus aureus	1	0.8%
Streptococcus spp.	3	2.5%
Streptococcus viridans	3	2.5%
Other gram-positive bacteria	2	1.7%
Aerobe gram-negative bacteria	54	44.6%
Enterobacteriacae	52	43.0%
Enterobacter spp.	16	13.2%
Escherichia coli	13	10.7%
Klebsiella spp.	12	9.9%
Citrobacter spp.	9	7.4%
Non-Fermenters	4	3.3%
Pseudomonas aeruginosa	2	1.7%
Stenotrophomas maltophilia	2	1.7%
Other gram-negative bacteria	1	0.8%
Anaerobe bacteria	4	3.3%
Clostridium perfingens	4	3.3%
Fungi	12	9.9%
Candida spp.	11	9.1%
Saccharomyces cervisiae	1	0.8%

**Table 4:** Interventional therapy of initial hospital stay and interventional therapy considering all follow-ups (CT: CT-guided puncture, ERC: endoscopic retrograde cholangiographic guided puncture and stenting, PTCD: percutaneous transhepatic cholangiodrainage, SGP sonographic-guided puncture)

Therapy	Number of patients during initial hospital stay	Proportion of patients (%) during initial hospital stay	Number of patients during clinical follow-up	Proportion of patients (%) during clinical follow-up
Wait-and-see approach				
No intervention	7	21.9%	6	18.8%
Single modal therapy				
CT	1	3.1%	2	6.3%
ERC	5	15.6%	5	15.6%
PTCD	1	3.1%	1	3.1%
Multimodal therapy				
ERC, SGP	3	9.4%	2	6.3%
ERC, CT	7	21.9%	5	15.6%
ERC, SGP, CT	1	3.1%	1	3.1%
PTCD, SGP	2	6.3%	2	6.3%
PTCD, CT	1	3.1%	1	3.1%
Rendezvous, SGP	2	6.3%	1	3.1%
Rendezvous, SGP, CT	0	0.0%	1	3.1%
ERC, PTCD (no rendezvous)	1	3.1%	1	3.1%
ERC, PTCD, CT	1	3.1%	2	6.3%
ERC, SGP, CT, PTCD	0	0.0%	1	3.1%
Surgery				
Biloma abscess resection	0	0.0%	1	3.1%

**Table 5:** Complications of interventional therapy

Complication of interventional therapy	ERC (*n* = 147)	PTCD (*n* = 41)	Rendezvous (*n* = 11)
Cholangitis	2 (1.4%)	2 (4.9%)	1 (9.1%)
Pancreatitis	1 (0.7%)	–	–
Bleeding	2 (1.4%)	–	–
Perforation	–	1 (2.4%)	–

### Microbiological analysis of bilomas

According to the Standard Operating Procedure (SOP) of our department of gastroenterology, bile collection is only performed if infection of the biliary tract system is suspected. Overall, biloma infection was suspected in 25 patients. Therefore, bile collection was performed in 25/32 (78.1%) patients. However, according to our study criteria for infected biloma, 10/25 (40%) proved to suffer from infected biloma. This subgroup revealed positive microbiology in 8 cases and sterile microbiology in 2 cases. 15/25 patients with bile culture (60%) did not fulfil our study criteria for infected biloma, mainly because a concurrent infection focus like urinary tract infection or pneumonia was diagnosed or could not be ruled out. None of these bile cultures were sterile. No significant difference in pathogen spectrum between infected and colonized biloma was found.

46 out of 49 bile cultures (93.9%) were positive. In total, 121 microbiologic organisms could be isolated. The group of *Enterobacteriacae* was most frequently isolated (43.0%, 52/121), followed by *Enterococcus* spp. (32.2%, 39/121) and *Candida* spp. (9.1%, 11/121) as shown in Table 3. Analysis of antimicrobial susceptibility testing revealed that 25.0% of our study population (8/32) presented biloma infection by *Enterobacteriaceae* with a Third-Generation-Cephalosporine resistance (ESBL/AmpC, 8/32) or by Vancomycin resistant *Enterococci faecium* (VRE, 1/32).

### Treatment

Table 4 displays drainage approaches including ERC, PTCD, CT- or sonographic-guided puncture (SGP) and rendezvous intervention. Median size of biloma in patients with intervention was 5.85 cm (range: 2–19.5 cm) compared to 4.3 cm (range: 2.5–7.9 cm, *P* = 0.1996) without intervention. 8/26 patients received a single modal therapy. 18/26 patient underwent multimodal therapy combining two or more different interventional approaches.

The overall complication rate of all interventions was 4.8% (9/188), see Table 5. Main complications included occult perforation after PTCD (which was conservatively treated), cholangitis, bleeding and mild pancreatitis. CT- and sonographic guided puncture were not associated to complications in the course of this study. In case of biliary and extra-biliary infections patients received antimicrobial treatment. The most frequently used antibiotic was Ciprofloxacin (18/32, 56.2%), followed by Piperacillin/tazobactam (11/32, 34.4%).

### Follow-up

Follow-up analyses revealed complete resolution of bilomas in 78.3% in the intervention group (18/23, follow-up median: 45 days, range 20–530 days). The non-interventional group presented a resolution rate of 80% (4/5; follow-up median: 19 days, range 6–55 days). 2 patients of the intervention group and 2 patients of the wait and see group were lost to follow-up. 6 patients died during the follow-up period. Patients deceased due to end-stage malignancies (3/6) or due to non-biloma associated septic complications after liver transplantation (3/6). One patient was converted to surgery after failure of CT-, and ERC-guided drainage.

## Discussion

In the current study, aetiology, clinical presentation, spectrum of pathogens, therapy options and therapy complications of biloma are retrospectively investigated in a group of 32 patients. In line with the present study, the majority of biloma cases are described after hepatobiliary surgery: 65.6% of patients developed one or more biloma after liver transplantation, cholecystectomy or after hepatic resection. Guillaud et al. retrospectively screened 1001 patients undergoing hepatic resection for post-operative complications and found an incidence of bilomas of 4.2% (42/1001) [11]. Safdar et al. [7] investigated the occurrence rate of biloma after liver transplantation. Of the 492 patients who received liver transplantation, 57 patients (11.6%) developed a biloma after a median interval of 9.1 weeks (2–377 weeks). In our study, median time to diagnosis was 8.6 weeks (range 2 weeks - 31.1 months) after liver transplantation. Concerning non-surgical aetiologies, most biloma developed after endoscopic retrograde cholangiography (ERC). In literature, incidence of biliary leakage of patients undergoing ERC is less than 0.5% [12, 13]. Dependent on the extent of the biliary injury, biliary leakage can either close spontaneously or lead to the development of biloma. Furthermore, 2 patients developed biloma after TACE and one after photodynamic treatment for cholangiocelluar carcinoma. In a former study by Zhang et al. [14], complications rate of 1923 patients with 4695 TACE procedures was analysed. The incidence of biloma was approximately 1% in this study. Few cases include the occurrence of biloma after spontaneous stone-filled gallbladder perforation [10], after alithiasic cholecystitis [15] or post traumatic as described after a stab wound for one of the patients of our present study.

Corresponding to the literature, icterus, fever and abdominal pain were the leading clinical symptoms [7–9, 16]. One third of biloma patients were asymptomatic as already reported by Safdar et al. [7]. Symptomatic patients in our study had a significantly longer hospital stay duration than asymptomatic patients. However, the size of bilomas seems not to be decisive for symptoms or clinical course. Nevertheless, patients with multiple bilomas presented more frequently clinical symptoms compared to patients with only one biloma, although this difference was not significant.

The pathogen spectrum of bilomas resembles the cholangitis’ pathogen spectrum. The group of *Enterobacteriacae* and *Enterococcus* spp. were the most frequent pathogens isolated from bilomas. In a previous study [17], we analysed the spectrum of pathogens of 447 cholangitis episodes: *Enterococcus* spp. followed by *E. coli* and *Klebsiella* spp. were the most frequently isolated pathogens. Other studies analysing the pathogen spectrum of acute cholangitis also found the group of *Enterobacteriaceae* and *Enterococcus* the most common pathogens in acute cholangitis [18–21]. Concerning antimicrobial resistance, the current data showed a relatively high proportion of multiresistant bacteria. In 8/32 patients *Enterobacteriaceae* with a Third-Generation-Cephalosporine resistance (ESBL/AmpC) or Vancomycin resistant *Enterococcus faecium* were isolated. The high incidence of multiresistant bacteria might be related to the high proportion of seriously ill patients. 7 patients developed bilomas after liver transplantation, 13 patients suffered from tumour disease and 6 patients deceased during the follow-up due to severe pre-existing conditions. No patients died due to infected biloma. In the setting of oncologic patients or patients undergoing surgery, susceptibility to Health care associated infections seems to be prominent [22, 23]. Thus, probability of antimicrobial treatment in this patient group is higher, promoting antimicrobial resistance.

Interventional drainage approaches of bilomas including ERC, PTCD, CT- or sonographic-guided puncture (SGP) and rendezvous intervention seems to be safe and effective. Follow-up analyses revealed complete resolution of bilomas in 78.3% in the intervention group. The overall complication rate was 4.8%. The majority of complications had a mild course. Perforation occurred in one patient, who was conservatively treated. Only one patient had to be converted to surgery.

Limitations of this study include the recruitment of patients based on an endoscopic database and hereby selecting more patients with extensive morbidity, which may have influenced the generalizability of the results of the study. Patients with extensive (co)morbidities are more prone to infections, may receive more frequently antibiotic treatment and therefore have a higher risk of acquiring multiresistant bacteria than patients with less comorbidities. However, one major aim of the study was to show that endoscopic treatment is safe and efficient for patients with bilomas. Furthermore, due to the high proportion of patients with antibiotic treatment initiated either because of a concurrent, non-biloma associated infection focus (i.e. pneumonia, urinary tract infection) or prophylactic reasons (i.e. endocarditis prophylaxis), the incidence of infected biloma might be underestimated in our study cohort. Further drawbacks of the study are its single-centre character and its retrospective study design.

## Conclusion

Major focus of the current trial was to analyse aetiology, clinical presentation, pathogen spectrum, and treatment of patients with bilomas. Two thirds of the patient cohort underwent surgery before developing bilomas (i.e. liver transplantation, cholecystectomy or partial liver resection). One third of the study population developed bilomas due to a non-surgical aetiology (i.e. TACE, photodynamic therapy or ERC). Main clinical symptoms of patients with bilomas were icterus, fever, and abdominal pain. The pathogen spectrum of bilomas was similar to the spectrum of acute cholangitis. *Enterococcus* spp. and *Enterobacteriacae* were the predominant pathogens isolated from bilomas. Analysis of antimicrobial susceptibility testing revealed a relatively high proportion of multiresistant bacteria. Finally, several interventional drainage approaches were used for treatment of bilomas, which all seem to be safe and effective.

## Abbreviations

ERC: Endoscopic retrograde cholangiography
GOT: Glutamic oxaloacetic transaminase
MALDI-TOF: Matrix-associated laser desorption/ionization-time of flight
PTCD: Percutaneous transhepatic cholangiodrainage
SGP: Sonographic guided puncture
SOP: Standard Operating Procedure
TACE: Trans arterial chemoembolization

## References

[1] Gould L, Patel A (1979). Ultrasound detection of extrahepatic encapsulated bile: "biloma". AJR Am J Roentgenol.

[2] Hajjar NA, Tomus C, Mocan L, Mocan T, Graur F, Iancu C (2014). Management of bile duct injuries following laparoscopic cholecystectomy: long-term outcome and risk factors infuencing biliary reconstruction. Chirurgia (Bucharest, Romania : 1990).

[3] Gössling PAM, Alves GRT, Silva RVA, Missel JR, Marques HF, Haygert CJP (2012). Spontaneous biloma : a case report and literature review. Radiol Bras.

[4] Sayar S, Olmez S, Avcioglu U, Tenlik I, Saritas B, Ozdil K (2016). A retrospective analysis of endoscopic treatment outcomes in patients with postoperative bile leakage. Northern clinics of Istanbul.

[5] Fujiwara H, Yamamoto M, Takahashi M, Ishida H, Ohashi O, Onoyama H (1998). Spontaneous rupture of an intrahepatic bile duct with biloma treated by percutaneous drainage and endoscopic sphincterotomy. Am J Gastroenterol.

[6] Lubbert C, Wiegand J, Karlas T. Therapy of liver abscesses. Viszeralmedizin 2014, 30(5):334–341. 10.1159/000366579 PMC4513824 26287275

[7] Safdar N, Said A, Lucey MR, Knechtle SJ, D’Alessandro A, Musat A (2004). Infected bilomas in liver transplant recipients: clinical features, optimal management, and risk factors for mortality. Clin Infect Dis.

[8] Bas G, Okan I, Sahin M, Eryilmaz R, Isik A (2011). Spontaneous biloma managed with endoscopic retrograde cholangiopancreatography and percutaneous drainage: a case report. J Med Case Rep.

[9] Lee JH, Suh JI (2007). A case of infected biloma due to spontaneous intrahepatic biliary rupture. The Korean journal of internal medicine.

[10] Taneja S, Sharma A, Duseja AK, Kalra N, Chawla Y (2011). Spontaneous perforation of gallbladder with intrahepatic bilioma. Journal of clinical and experimental hepatology.

[11] Guillaud A, Pery C, Campillo B, Lourdais A, Sulpice L, Boudjema K (2013). Incidence and predictive factors of clinically relevant bile leakage in the modern era of liver resections. HPB : the official journal of the International Hepato Pancreato Biliary Association.

[12] Enns R, Eloubeidi MA, Mergener K, Jowell PS, Branch MS, Pappas TM (2002). ERCP-related perforations: risk factors and management. Endoscopy..

[13] Vandervoort J, Soetikno RM, Tham TC, Wong RC, Ferrari AP, Montes H (2002). Risk factors for complications after performance of ERCP. Gastrointest Endosc.

[14] Zhang B, Guo Y, Wu K, Shan H (2017). Intrahepatic biloma following transcatheter arterial chemoembolization for hepatocellular carcinoma: incidence, imaging features and management. Mol Clin Oncol.

[15] Pinilla Fernandez I, Marti de Gracia M, de Agueda Martin S (2005). Acute post-cholecystitis bilioma. Based on two cases. Revista clinica espanola.

[16] Della Valle V, Eshja E, Bassi EM (2015). Spontaneous biloma: a case report. Journal of ultrasound.

[17] Weber A, Schneider J, Wagenpfeil S, Winkle P, Riedel J, Wantia N, et al. Spectrum of pathogens in acute cholangitis in patients with and without biliary endoprosthesis. J Inf Secur. 2013. 10.1016/j.jinf.2013.04.008 23603487

[18] Leung JW, Ling TK, Chan RC, Cheung SW, Lai CW, Sung JJ (1994). Antibiotics, biliary sepsis, and bile duct stones. Gastrointest Endosc.

[19] Leung JW, Liu YL, Lau GC, Chan RC, Lai AC, Ling TK (2001). Bacteriologic analyses of bile and brown pigment stones in patients with acute cholangitis. Gastrointest Endosc.

[20] Brook I (1989). Aerobic and anaerobic microbiology of biliary tract disease. J Clin Microbiol.

[21] Schneider J, Hapfelmeier A, Fremd J, Schenk P, Obermeier A, Burgkart R (2014). Biliary endoprosthesis: a prospective analysis of bacterial colonization and risk factors for sludge formation. PLoS One.

[22] Wang FD, Chen YY, Chen TL, Liu CY (2008). Risk factors and mortality in patients with nosocomial Staphylococcus aureus bacteremia. Am J Infect Control.

[23] Yang Z, Wang J, Wang W, Zhang Y, Han L, Zhang Y (2015). Proportions of Staphylococcus aureus and methicillin-resistant Staphylococcus aureus in patients with surgical site infections in mainland China: a systematic review and meta-analysis. PLoS One.

